# The Clinical Significance of SIRT2 in Malignancies: A Tumor Suppressor or an Oncogene?

**DOI:** 10.3389/fonc.2020.01721

**Published:** 2020-09-08

**Authors:** Lin Zhang, Sungjune Kim, Xiubao Ren

**Affiliations:** ^1^Department of Immunology, Tianjin Medical University Cancer Institute and Hospital, Tianjin, China; ^2^National Clinical Research Center of Cancer, Tianjin, China; ^3^Key Laboratory of Cancer Immunology and Biotherapy, Tianjin, China; ^4^Department of Radiation Oncology and Immunology, H. Lee Moffitt Cancer Center and Research Institute, Tampa, FL, United States; ^5^Department of Biotherapy, Tianjin Medical University Cancer Institute and Hospital, Tianjin, China

**Keywords:** sirtuins, sirtuin 2, tumor suppressor, oncogene, HDACs

## Abstract

Sirtuin 2 (SIRT2) is a member of the sirtuin protein family. It is a Class III histone deacetylase (HDACs) and predominantly localized to the cytosol. SIRT2 deacetylates histones and a number of non-histone proteins and plays a pivotal role in various physiologic processes. Previously, SIRT2 has been considered indispensable during carcinogenesis; however, there is now a significant controversy regarding whether SIRT2 is an oncogene or a tumor suppressor. The purpose of this review is to summarize the physiological functions of SIRT2 and its mechanisms in cancer. We will focus on five malignancies (breast cancer, non-small cell lung cancer, hepatocellular carcinoma, colorectal cancer, and glioma) to describe the current status of SIRT2 research and discuss the clinical evaluation of SIRT2 expression and the use of SIRT2 inhibitors.

## Introduction

Posttranslational modifications fine tune the biological activity of many proteins (1, 2). In recent years, there has been a significant interest in the role of protein acetylation (3). Sirtuins are protein deacetylases, including a family of proteins (SIRT1–7) with homology to the silent information regulator 2 (Sir2) gene in *Saccharomyces cerevisiae* (4, 5). This family of proteins contains highly conserved enzymes categorized as Class III histone deacetylases (HDACs III), and their deacetylase activity is dependent on nicotinamide adenine dinucleotide (NAD) as a cofactor distinct from zinc-dependent HDACs (6, 7).

Sirtuins are heterogeneous in their subcellular locations, which reflect their broad range of biological functions. SIRT1 is mainly a nucleoprotein, although it can also be found in the cytoplasm (8). SIRT3–5 are constitutively localized to the mitochondria (9). SIRT6 and SIRT7 are also predominantly in the nucleus (10, 11). Sirtuin 2 (SIRT2) is the only sirtuin predominantly found in the cell cytoplasm (7); however, it can shuttle in and out of its primary location, using mechanisms which may be cell and tissue dependent (8, 12). Consistent with its predominant cytosolic location, SIRT2 deacetylates a number of non-histone proteins. Last decade, many new substrates and SIRT2-related proteins had been identified, such as CDK9, PGAM2, Par-3, and CDH1/CDC20, etc. (Table 1) (13–25). These results suggest that SIRT2 regulates multiple biological functions, including neurotoxicity, metabolism, mitosis regulation, genome integrity, oxidative stress, and autophagy (Table 1). Currently, there is growing evidence that abnormal expression of SIRT2 is primarily associated with two human diseases, neurologic diseases, and cancer.

**TABLE 1:** The deacetylase substrates of SIRT2.

Substrates	Site	Mechanism	Functions	References
Par-3 AMPAR α-syn	– K813/819/822/868 K6/10	Active aPKC Degradation Aggregate	Neurogenesis (myelination) Neurogenesis (synaptic plasticity) Neurotoxicity	Beirowski et al. (13) Wang et al. (14) de Oliveira et al. (15)
FOXO1 ACLY PEPCK1	– K540/546/554 K70/71/594	Inhibition Degradate Stability	Metabolism (Adipogenesis) Metabolism (lipogenesis) Metabolism (Gluconeogenesis)	Jing et al. (16) Lin et al. (17) Jiang et al. (18)
CDH1/CDC20 PR-Set7	K69/159, K66 K90	Activate APC/C Chromatin localization	Mitosis regulation Mitosis regulation	Kim et al. (19) Serrano et al. (20)
CDK9 ATRIP	K48 K32	Activate Activate ATR	Genome integrity Genome integrity	Zhang et al. (21) Zhang et al. (22)
PGAM2 G6PD	K100 K403	Activate Activate	Oxidative stress Oxidative stress	Xu et al. (23) Wang et al. (24)
FOXO1	K262/265/274	Unbound to ATG7	Autophagy	Zhao et al. (25)

To date, the role of SIRT2 in malignancy has attracted widespread attention, but it is still under debate. There are two opposing viewpoints that support SIRT2 functioning as an oncogene and a tumor suppressor. Based on the existing research, this review summarizes the physiological functions of SIRT2 and its mechanisms in cancer. We will focus on five malignancies, breast cancer, non-small cell lung cancer, hepatocellular carcinoma (HCC), colorectal cancer, and glioma, in which the pattern of SIRT2 expression and its physiologic functions are controversial.

## Physiologic Functions of SIRT2

### SIRT2 and the Nervous System

Among all sirtuins, SIRT2 is the most highly expressed in brain tissue, particularly in the cortex, striatum, spinal cord, and postnatal hippocampus (26, 27), indicating that SIRT2 is involved in neural development. Several studies have reported that SIRT2 is crucial for myelination whether in the central nervous system (CNS) or peripheral nervous system. In the CNS, SIRT2 is mainly expressed in oligodendrocytes (OLs) and is considerably upregulated during OL differentiation and myelination (28, 29). In the peripheral nervous system, SIRT2 ablation in mouse Schwann cells (SCs) delayed myelin formation and postinjury remyelination (13, 30). Moreover, SIRT2 is involved in other developmental processes in the nervous system as SIRT2 gene knockout mice demonstrated dysfunctions of the nervous system, such as defects in differentiation of dopaminergic (DA) neurons (31), aberrant synaptic plasticity with impaired learning and memory (14), and morphological changes of mitochondria in the cortex (32).

In addition to act as a crucial regulator in neurodevelopment, SIRT2 is also associated with nervous system disorders, in particular, neurodegenerative diseases [Parkinson’s disease (PD), Alzheimer’s disease (AD), and Huntington’s disease (HD)] (33). SIRT2 expression participates in the aggregation process of proteins such as α-synuclein (α-syn) (15), huntingtin (34), as well as amyloid-β peptide (Aβ), and hyperphosphorylated tau protein (35, 36), involved in PD, HD, and AD, respectively. Mounting evidence showed that inhibition of SIRT2 function, either pharmacologically or genetically, provided neuroprotection in a variety of mice modals, suggesting that SIRT2 could be a potential therapeutic target for these diseases (37–39). The association between SIRT2 and neuromalignance will be discussed.

### SIRT2 and Metabolism

The potential roles and effects of SIRT2 to maintain metabolic homeostasis have been recognized more recently. SIRT2 is the most prominently expressed sirtuin in the adipose tissue both *in vivo* and in culture (40), implicating its involvement in lipid metabolism, adipogenesis, lipid synthesis, and fatty acid oxidation. In mouse 3T3-L1 preadipocytes, SIRT2 deacetylates the nuclear transcription factor FOXO1, which results in nuclear retention of this protein where it represses the transcription of PPARγ (peroxisome proliferator-activated receptor γ), culminating in the inhibition of adipocyte differentiation (16). Another study showed the role of SIRT2 in the regulation of lipid synthesis. Under high-glucose conditions, SIRT2 deacetylates ATP-citrate lyase (ACLY), a lipogenic enzyme, leading to its ubiquitylation, and degradation (17). Krishnan and colleagues showed that fat cell-specific HIF-1α inactivation in obese mice causes accumulation of nuclear SIRT2, which deacetylates PGC-1α, thereby, promoting fatty acid oxidation (41).

Regarding glucose metabolism, SIRT2 regulates gluconeogenesis via deacetylating PEPCK1, which catalyzes the first rate-limiting step of gluconeogenesis. When glucose level is high, PEPCK1 acetylation increases, which promotes its interaction with the UBR5 E3 ubiquitin ligase and proteosomal degradation, thus, suppressing gluconeogenesis. In contrast, when glucose level is low, PEPCK1 is deacetylated by SIRT2, and its stabilization enhances gluconeogenesis (18).

### SIRT2 and the Cell Cycle

Sirt2 colocalizes with microtubules and deacetylates α-tubulin (7). However, during the G2/M transition of the cell cycle, Sirt2 can transiently migrate to the nucleus to deacetylate histone H.4 lysine 16 (H4K16Ac) (8, 12), thereby regulating chromosomal condensation during mitosis. Moreover, cells with SIRT2 overexpression exhibit marked prolongation of the mitotic phase *in vitro* (12, 42). These results suggest a role for SIRT2 in regulating mitotic processes.

Investigation of *Sirt2^–/–^* mice ultimately uncovered the mechanism via which SIRT2 regulates mitosis. *Sirt2^–/–^* cells displayed widespread centrosome amplification and aneuploidy, which resulted in genetic instability and abnormal mitosis both *in vitro* and *in vivo* (19, 20). Indeed, Kim et al. reported that anaphase-promoting complex/cyclosome (APC/C), an E3 ubiquitin ligase with multiple subunits, which mediates ubiquitination of key regulators of the cell cycle, is positively regulated by SIRT2 through deacetylation of its coactivators, CDH1, and CDC20 (19). SIRT2 deficiency causes hyperacetylation of CDH1 and CDC20, impaired activity of APC/C, and hence upregulation of Aurora-A levels, which consequently lead to abnormalities in mitosis. Serrano et al. provided another possible mechanism (20). SIRT2 deacetylation of K90 residue of PR-Set7 modulates its chromatin localization. Consistently, SIRT2 depletion significantly reduced PR-Set7 chromatin levels, altering the size and number of PR-Set7 foci to decrease the overall mitotic deposition of H4K20me1.

Furthermore, other studies have shown that SIRT2 regulates genome integrity through deacetylation of CDK9 (21) or ataxia telangiectasia-mutated and Rad3-related (ATR)-interacting protein (ATRIP) (22). Overall, the crucial role of SIRT2 in mitosis regulation and genome integrity implies that its activity may have a significant effect on cancer, a disease with high genetic instability and abnormal mitosis (19).

## SIRT2 and Cancer

In recent years, a growing body of evidence has proposed a role for SIRT2 in tumorigenesis. Because SIRT2 is expressed in a wide range of tissues and organs and exerts variable physiological functions, its role in cancers is complicated. Notably, SIRT2 has been described as both an oncogene and a tumor suppressor. In this review, we will discuss the divergent expression and function of SIRT2 in five malignancies: breast cancer, non-small cell lung cancer, HCC, colorectal cancer, and glioma.

### Breast Cancer

#### Molecular Pathways Targeted by SIRT2

The role of SIRT2 in tumorigenesis has been extensively studied in breast cancer. Kim et al. observed a significant propensity of *Sirt2^–/–^* female mice to develop mammary tumors at an old age, suggesting a role for SIRT2 as a tumor suppressor (19). As discussed earlier, a possible mechanism proposed for this phenotype is Sirt2 regulation of APC/C activity through CDH1 and CDC20. SIRT2 deficiency causes increased levels of Aurora-A and, consequently, contribute to centrosome amplification, aneuploidy, genomic instability, mitotic cell death, and most importantly, spontaneous tumor formation. Serrano et al. used a skin papilloma model to further study the role of SIRT2 in tumorigenesis (20). Following DMBA/TPA treatment on the skin, *Sirt2^–/–^* mice developed larger papilloma at higher frequencies than wild-type mice. Histopathological study demonstrated that most of the papilloma developed into squamous cell carcinoma and fibrosarcoma.

Park et al. further investigated SIRT2 function using *Sirt2^–/–^* mammary tumor cell line (MMT) derived from the spontaneous mammary tumors in *Sirt2^–/–^* mice (43). They identified the M2 isoform of pyruvate kinase (PKM2) as a critical target of SIRT2. Indeed, loss of SIRT2 altered PKM2 activity and reprogrammed glycolytic metabolism in cancer cells, which was associated with increased tumorigenesis in *Sirt2^–/–^* mice.

Although genetic studies tend to suggest that SIRT2 is a tumor suppressor, pharmacological studies suggest the opposite. Jing et al. developed a potent SIRT2-specific inhibitor, TM (a thiomyristoyl lysine compound), which demonstrated a broad anticancer activity, including activity against several breast cancer cell lines. The study proposed that SIRT2 inhibition promotes NEDD4 expression, an E3 ubiquitin ligase for c-Myc, and thus causing c-Myc ubiquitination and degradation (44). Shah et al. developed another inhibitor of SIRT2 (RK-9123016), which also reduced the viability of human breast cancer cells via downregulated c-Myc expression. This drug was found to increase the acetylation level of eIF5A (eukaryotic translation initiation factor 5A), another physiological substrate of SIRT2 (45).

Similarly, studies focused on CSCs (cancer stem cells) and BLBC (basal-like breast cancer) suggest SIRT2 as an oncogene. CSCs are believed to contribute to tumor metastasis and poor prognosis. Notably, Zhao et al. demonstrated that SIRT2 protein levels were significantly increased in ALDH1^+^ CSCs isolated from primary human breast tumors compared with the levels in ALDH1^–^ cells. In addition, they demonstrated NOTCH-induced SIRT2 deacetylation activity on K353 of ALDH1A1, which led to its enzymatic activation to maintain breast CSCs (46).

Basal-like breast cancer represents one of the most aggressive breast cancer subtypes characterized by increased propensity for metastasis and poor prognosis. SIRT2 has been reported to be frequently amplified and highly expressed in BLBC. Slug protein has been found to be a deacetylase target of SIRT2, and SIRT2 overexpression promoted Slug stability, thus, conferring aggressive, basal-like malignant features, and growth. By contrast, genetic depletion and pharmacological inactivation of SIRT2 in BLBC cells reversed Slug stabilization and, thus, abrogated relevant pathological features of BLBC and inhibited tumor growth (47). Another study using MDA-MB-231 cell (a BLBC cell line) demonstrated that SIRT2 silenced Arrestin domain-containing 3 (ARRDC3), a tumor suppressor, contributing to the aggressive nature of BLBC cells (48).

**FIGURE 1:**
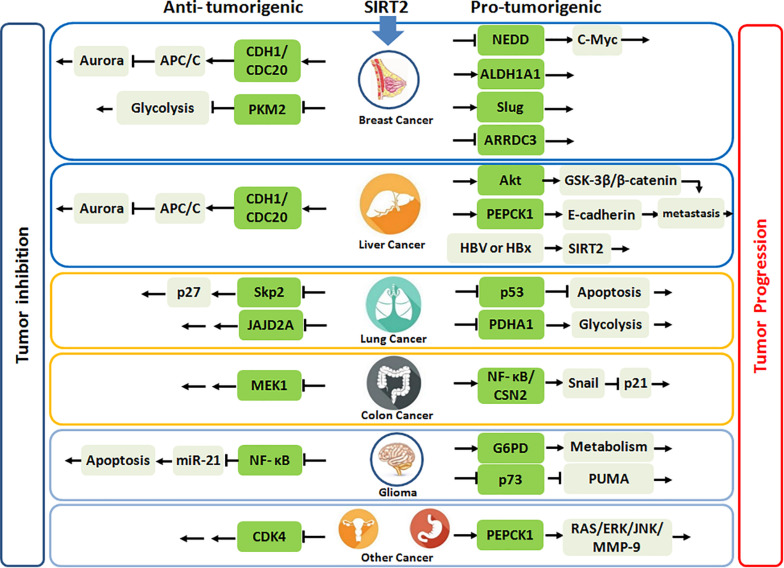
SIRT2 plays the anti-tumorigenic or pro-tumorigenic roles in various malignances. Highlighted green frames represent substrates or partners of SIRT2.

Taken together, SIRT2 may have a significant tumor suppressive role during early carcinogenesis of breast cancer, but conversely in advanced cancer, its overexpression portends more aggressive phenotype, and SIRT2 inhibition has anticancer activities. Importantly, SIRT2 functions may evolve from tumor initiation to progression with altered physiologic targets (Figure 1).

#### SIRT2 Expression and the Clinical Outcome

Several studies focused on the clinical relevance of SIRT2 expression in breast carcinoma. Following the initial studies with *Sirt2^–/–^* mice, Kim et al. investigated SIRT2 expression levels in human breast cancer samples. Using a tissue array with 36 paired samples of breast cancer and adjacent normal breast tissue, they demonstrated significantly higher levels of SIRT2 in normal breast tissues compared with cancer tissues. Furthermore, lower SIRT2 expression was observed in metastatic samples suggesting that SIRT2 downregulation may be associated with more aggressive phenotype in breast cancer. This result was supported by Shi et al. who demonstrated that high expression of SIRT2 by IHC (IHC score > 3) was lower in tumor tissues compared to the normal adjacent tissues in 296 patients (49). Similarly, McGlynn et al. detected that SIRT2 transcripts were significantly lower in malignant breast tissues in comparison to non-malignant or normal breast tissue (50). These studies suggested that SIRT2 may act as a tumor suppressor in breast cancer.

Although SIRT2 expression was lower in breast cancer than in normal tissue, residual SIRT2 expression was generally observed. In Kim’s study (19), among 36 cancer tissues tested, 66.7% of the tissues maintained low or intermediate levels of SIRT2 expression. Interestingly, when McGlynn et al. investigated the correlation between SIRT2 expression levels in breast cancer and prognosis using IHC staining in 153 ER^–^ and 392 ER^+^ breast cancer tissues, they observed more aggressive breast cancer phenotype with higher nuclear levels of SIRT2 (50). Indeed, in the ER^–^ cohort with approximately 80% grade 3 tumors, they observed that high SIRT2 nuclear levels were associated with shorter time to relapse and death compared to low SIRT2 nuclear expression. Similar results also emerged in grade 3 tumors of ER^+^ cohort. These results highlight that SIRT2 nuclear expression is associated with poor prognosis in advanced breast cancer.

**FIGURE 2:**
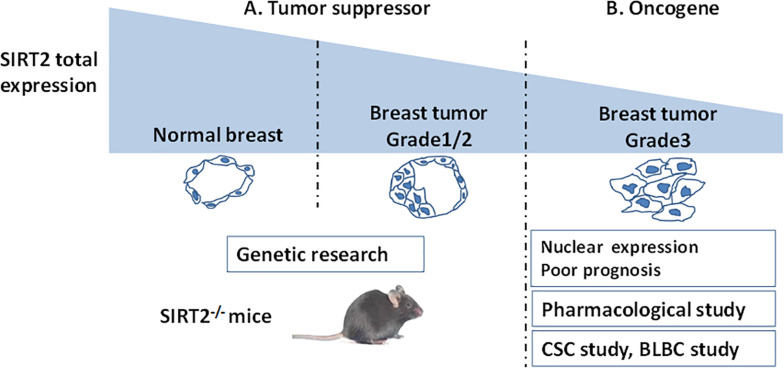
A schema shows SIRT2’s functions evaluated by different breast cancer studies. **(A)** SIRT2 is highly expressed in the normal breast compared with breast tumor. This clinical result suggest that SIRT2 might act as a tumor suppressor, which is supported by Genetic research (SIRT2 Knockout). **(B)** However, in Grade 3 breast cancer, high levels of nuclear SIRT2 are associated with poor clinical outcome, indicating that SIRT2 might act as an oncogene in more aggressive malignancies. The studies on CSC and BLBC support this inference. Pharmacological research of SIRT2 specific inhibitor also finds the oncogenic role of SIRT2.

Taken together, a lower expression of SIRT2 in breast cancer compared with that in normal breast indicates that SIRT2 might act as a tumor suppressor at the initiation of tumorigenesis. However, higher SIRT2 expression in advanced tumor tissues portend poor prognosis underscoring that SIRT2 may act as an oncogene during tumor progression (Figure 2 and Table 2).

**TABLE 2:** The clinical role of SIRT2 in five malignancies.

Malignance	Method of study	Role of SIRT2	References
Breast cancer	IHC IHC	Tumor suppressor Tumor suppressor	Kim et al. (19) Shi et al. (49)
	qPCR IHC	Tumor suppressor Oncogene (nuclear expression)	McGlynn et al. (50)
Hepatocellular carcinoma	Microarray qPCR, IHC IHC, database	Tumor suppressor Oncogene Oncogene	Kim et al. (19) Chen et al. (51) Huang et al. (52)
NSCLC	qPCR, WB, IHC IHC	Tumor suppressor Oncogene	Li et al. (54) Grbesa et al. (57)
Colorectal cancer	IHC WB, database	Tumor suppressor Oncogene	Zhang et al. (63) Hu et al. (67)
Glioma	qPCR, WB Northern blot	Tumor suppressor Tumor suppressor	Li et al. (70) Hiratsuka et al. (69)
	IHC	Oncogene (nuclear expression)	Imaoka et al. (73)

### Liver Cancer

#### Molecular Pathways Targeted by SIRT2

Kim et al. also reported increased the development of HCC in old male *Sirt2^–/–^* mice via similar mechanisms observed for breast cancer (19). However, Chen et al. demonstrated that depletion of SIRT2 in human HCC cell lines markedly reduced cell migration with a regression of epithelial–mesenchymal transition (EMT) phenotypes (51). Ectopic SIRT2 expression in L02 cell line was found to promote cell motility and invasiveness. Notably, SIRT2 deletion increased Akt acetylation, thereby, impairing Akt/GSK-3β/β-catenin-signaling cascade to regulate EMT and cell migration. In another study, Huang et al. also found that downregulation of SIRT2 reduced migration and invasion in human HCC cells, but revealed that SIRT2 inhibition increased PEPCK1 acetylation and suppressed its downstream E-cadherin pathway (52). Interestingly, Cheng et al. revealed that hepatitis B (HBV) or HBx upregulates SIRT2 expression by targeting its promoter, which then enhanced transformation of HBV-related HCC (53). Overall, these data suggest that SIRT2 upregulation is associated with malignant transformation in the liver (Figure 1).

#### SIRT2 Expression and the Clinical Outcome

Kim et al. also analyzed SIRT2 expression using a microarray containing 264 human HCC samples (19). They found that many HCCs demonstrated lower levels of SIRT2 than normal liver tissue. Contrary to this finding, Chen et al. found that SIRT2 mRNA is expressed at a similar level in tumor and adjacent tissues in 45 HCC samples (51). Interestingly, SIRT2 protein was found to be expressed at a higher level in tumors (23/45) based on Western blot (WB). Overexpression of SIRT2 in primary HCC tumors was associated with increased microscopic vascular invasion and poor prognosis. Huang et al. also found that SIRT2 was significantly increased in tumor tissues than in normal adjacent tissues in tissue microarrays containing 52 HCC samples. They also queried the TCGA database, which contained clinically annotated genomic data from 286 HCC samples, and found that a higher SIRT2 level in HCC was detrimental to patient survival (52). The data from the latter two studies with both mRNA and protein detection suggest that SIRT2 expression is a negative prognostic factor (Table 2).

### Lung Cancer

#### Molecular Pathways Targeted by SIRT2

Li et al. reported that overexpression of SIRT2 in A549 and H1299 cells was associated with delayed cell proliferation, increased apoptosis, and cell cycle arrest (54). The follow-up study revealed that overexpression of SIRT2 promoted Skp2 deacetylation and degradation, and eliminated the effect of Skp2 on p27, which resulted in an increase in p27 and suppression of NSCLC cell growth (55). Similarly Xu et al. demonstrated that SIRT2 bound to the promoter region of JMJD2A and negatively regulated JMJD2A expression, which led to the inhibition of NSCLC cell proliferation, colony formation, and tumor growth both *in vitro* and *in vivo* (56).

In contrast, Grbesa et al. reported that SIRT2 downregulation significantly decreased proliferation in NSCLC cell lines (57). Further, Hoffmann et al. identified two structurally related compounds, which selectively inhibited SIRT2, AEM1, and AEM2, and both sensitized NSCLC cells to etoposide, which damages DNA by targeting topoisomerase II. These inhibitors also increased p53 acetylation and activated p53-dependent apoptosis in NSCLC cells lines (58). Yang et al. developed N-(3-(phenoxymethylphenyl) acetamide derivatives as potent, selective SIRT2 inhibitors. Among the derivatives, 24A strongly restrained cell growth and suppressed NSCLC cell (H441) migration and invasion (59). Ma et al. combined dichloroacetic acid (DCA, a pyruvate dehydrogenase kinase inhibitor) with Sirtinol or AGK2 (SIRT2 inhibitors) to treat lung cancer cell lines and found that this combination produced a synergistic antitumor effect via activation of PDHA1 to shift metabolism from glycolysis to OXPHOS and enhance ROS generation and activation of AMPK signaling (60). Overall, there is significant discordance among studies on NSCLC, which may be related to the specific cellular contexts of NSCLC cell lines tested (Figure 1).

#### SIRT2 Expression and the Clinical Outcome

Li et al. evaluated SIRT2 expression in eight pairs of NSCLC and adjacent normal tissue samples by Q-PCR and WB, which demonstrated significantly lower expression of SIRT2 in tumor. This observation was reproduced in 53 paired NSCLC and normal lung tissue analyzed by microarray (54).

However, when Grbesa et al. evaluated SIRT2 protein levels in a cohort of 105 NSCLC patients using IHC, its expression, mostly confined within the cytosol, was significantly higher in primary tumors than in normal tissue. Further, patients with high levels of SIRT2 showed significantly shorter recurrence-free survival than patients with low levels (57).

These conflicting results may be due to histologic variability among NSCLC cells, which are subdivided into adenocarcinoma, squamous cell carcinoma, large-cell carcinoma, bronchoalveolar lung cancer, and mixed histologic types (e.g., adenosquamous) (61), with distinct biologic behaviors and outcome (62). Alternatively, multiple competing mechanisms may exist in NSCLC, which are preferentially elicited by SIRT2 depending on the cellular context (Table 2).

### Colon Cancer

#### Molecular Pathways Targeted by SIRT2

Zhang et al. reported that overexpression of endogenous SIRT2 reduced migration and invasion of SW480 cells. Blocking SIRT2 expression induced the proliferation and metastatic progression of HT29 cells (63). Bajpe et al. also illustrated SIRT2 as a determinant of response to EGFR inhibitors in colon cancer (64). SIRT2 can inhibit MEK1 activation by deacetylating MEK1 and keep a check on cell proliferation. In turn, loss of SIRT2 led to increased MEK1 acetylation and its kinase activity, thus, attenuating the response to upstream inhibition of either EGFR or BRAF. These results delineate SIRT2 as a tumor suppressor in CRC.

In contrast, Cheon et al. reported that treatment of human colon cancer cells with the SIRT2-specific inhibitor, AK-1, which inactivates the NFκB/CSN2 pathway to induce proteasomal degradation of Snail, upregulated p21 to induce G1 arrest and delayed proliferation (65). Farooqi et al. presented novel lysine-based thiourea compounds as potent and selective SIRT2 inhibitors that were less hydrophobic and easier to synthesize than TM (44), which potently inhibited tumor growth in an HCT116 xenograft murine model, supporting a role for SIRT2 as a viable therapeutic target for CRC (66) (Figure 1).

#### SIRT2 Expression and the Clinical Outcome

Zhang et al. revealed that SIRT2 was downregulated in CRC biopsy samples (*n* = 31, not paired) compared with the normal adjacent tissues (*n* = 26). SIRT2 immunostaining was largely localized to the cytoplasm of colonic epithelial cells. Interestingly, decreased SIRT2 expression was associated with adverse clinicopathological features and poor prognosis in colon cancer (63).

In contrast, Hu et al. utilized the Oncomine database to evaluate the expression of SIRT2 in CRC and found that the level of SIRT2 was higher in CRC tissues compared to the normal tissue samples (67). This finding was verified by protein expression using WB (*n* = 12) and IHC (*n* = 46). In the CRC dataset GSE24551, SIRT2 upregulation correlated with advanced TNM stage and a lower 5-year survival rate. A similar survival outcome was observed in another online database^1^.

Taken together, there are potentially opposite roles of SIRT2 in CRC. The limitation of these studies is the relatively low number of clinical specimens used. Also, online databases represent mRNA expression rather than protein levels (Table 2).

### Glioma

#### Molecular Pathways Targeted by SIRT2

Sirtuin 2 is highly expressed in brain tissue and plays a crucial role in the development of the nervous system and neurodegenerative diseases (68). In addition, SIRT2 is located at 19q13.2, a region known to be frequently deleted in human glioma (69), thereby indicating that SIRT2 may be a tumor suppressor in gliomas. Li et al. showed that Sirt2 overexpression reduced glioma cell proliferation and significantly activated proapoptotic proteins caspase 3 and Bax while inhibiting the antiapoptotic protein Bcl-2. Mechanically, Sirt2 deacetylated p65 at K310, and blocked p65 binding to the promoter region of miR-21, thus, suppressing the miR-21-modulated apoptosis pathway (70).

However, Ye et al. revealed that HSPB1 enhanced the binding between G6PD and SIRT2, which led to deacetylation and activation of G6PD, thus promoting cellular NADPH and pentose production in glioma cells and, thereby, protecting cells from oxidative and DNA damage stress (71). Further, Funato et al. demonstrated that SIRT2 deacetylated C-terminal lysine residues of p73 and inactivated its transcriptional activity, and SIRT2 inhibition in glioblastoma cell lines (GB2 or GB16) resulted in p73-mediated transactivation of PUMA and induction of apoptosis (72) (Figure 1).

#### SIRT2 Expression and the Clinical Outcome

Li et al. reported that SIRT2 mRNA and protein expression was downregulated in eight primary glioma samples (grade II *n* = 2, grade III *n* = 5, and grade IV *n* = 1) *versus* four normal brain tissue samples tested. Protein and mRNA levels of SIRT2 in five human glioma cell lines (T98G, U87MG, U251, A172, and CCF-STTG1) were also lower than two normal human astrocyte cell lines (NHA and HA) (70).

Using a 2D-proteomics technique, Hiratsuka et al. identified SIRT2 downregulation in glioma tissue when compared to normal adjacent tissue. Northern blot analysis also revealed that RNA expression of SIRT2 was dramatically diminished in 12 out of 17 gliomas. Ectopic expression of SIRT2 in glioma cell lines disrupted the microtubule network causing a remarkable reduction in colony formation (69). However, investigation of SIRT2 expression by IHC in samples from 23 patients with glioblastoma (grade IV), eight patients with diffuse astrocytoma (grade II), and five healthy individuals revealed that SIRT2 preferential nuclear localization was more frequent in the malignant specimens, which was positively correlated with malignant progression (73). Therefore, SIRT2 nuclear translocation may be associated with its oncogenic effects in glioma (Table 2).

### Other Cancers

Dysregulated SIRT2 expression has been observed in multiple other cancers with clear clinical relevance, including renal cell carcinoma (RCC), gastric cancer, and melanoma, while SIRT2 appears downregulated in ovarian carcinoma and prostate cancer (Figure 1). Wei et al. demonstrated that SIRT2 was highly expressed in the stem-like RCC cells with adverse clinical outcome (74). In gastric cancer, SIRT2 had been found to be upregulated compared to adjacent normal tissues and also correlated with poor patient survival. Mechanically, SIRT2 altered PEPCK1 activity, and mitochondrial respiration while inducing gastric cancer cell migration and invasion by activating the RAS/ERK/JNK/MMP-9 pathway (75). SIRT2 upregulation was also found in metastatic melanoma with a predominant nuclear staining (76).

In contrast, SIRT2 was significantly downregulated in serous ovarian carcinoma (SOC) when compared with ovarian surface epithelium. Downregulated SIRT2 failed to repress CDK4 expression, eventually leading to accelerated SOC cell proliferation (77). SIRT2 loss also correlated with aggressive prostate cancer. In multiple datasets, decreased SIRT2 expression portended worse clinicopathologic outcomes (78).

## Conclusion

The expression pattern of SIRT2 and its correlation with clinical outcome is variable with opposite reports (Table 2). Inconsistent results from clinical studies raise the utility of SIRT2 expression as a biomarker. To minimize variability, we suggest determining the subcellular localization of SIRT2 since nuclear or cytoplasmic expression may have different functions. Additionally, cancer subtypes should be fully considered as SIRT2 might have distinct functions in different oncopathological conditions. Because of variability involved, clinical studies evaluating SIRT2 require large sample sizes, selection of highly specific antibodies, and preferably multicenter collaboration.

The plethora of SIRT2 substrates identified reflect cell or tissue-specific functions of SIRT2, which may evolve during different phases of malignant transformation. The role of SIRT2 in cancer is thus complex with multiple competing mechanisms, and therefore, SIRT2 cannot be simply considered as an oncogene or a tumor suppressor.

## Author Contributions

LZ and XR conceived and designed the review. LZ wrote the manuscript. SK reviewed and edited the manuscript. All authors read and approved the manuscript.

## Conflict of Interest

The authors declare that the research was conducted in the absence of any commercial or financial relationships that could be construed as a potential conflict of interest.

## Footnotes

1 https://www.proteinatlas.org/
